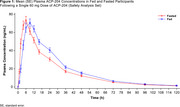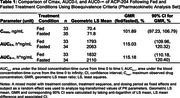# Effect of Food Consumption on the Pharmacokinetics of ACP‐204, a Novel 5‐HT_2A_ Receptor Selective Antagonist/Inverse Agonist

**DOI:** 10.1002/alz70859_105644

**Published:** 2025-12-26

**Authors:** Mona Darwish, Bryan Dirks, Xiaoshu Feng, Brian Raether, Sanjeev S Pathak

**Affiliations:** ^1^ Acadia Pharmaceuticals Inc., Princeton, NJ USA

## Abstract

**Background:**

ACP‐204 is an investigational drug with selective inverse agonism/antagonism of 5‐HT_2A_ receptors. Food‐drug interactions can alter the pharmacokinetics (PK) and pharmacodynamics of drugs, impacting their therapeutic efficacy and safety. This study evaluated the effect of concomitant food intake on the PK and safety of ACP‐204 in healthy participants.

**Method:**

In this single‐center, phase 1, randomized, open‐label, crossover study, participants received a 60‐mg oral capsule of ACP‐204 in the morning while either (A) fasted overnight (reference) or (B) following a high‐fat meal. Participants were randomized (1:1) to either treatment sequence AB or BA. PK sampling occurred on Days 1‐6 after study drug administration, followed by a 3‐day washout period before crossover. The extent of absorption was assessed by area under the blood or plasma concentration‐time curve (AUC); rate of absorption was assessed by the maximum drug concentration (C_max_). Each parameter was analyzed using a mixed effects general linear model. Safety was evaluated throughout the study including treatment‐emergent adverse events (TEAEs), clinical examinations, and laboratory tests.

**Result:**

A single 60‐mg dose of ACP‐204 was administered to 36 participants, with an average age of 39.1 years and 19.4% being female. ACP‐204 plasma concentrations are shown in Figure 1. The 90% CI for the geometric mean ratio (GMR) of fasted to fed for AUC_0‐∞_ (GMR=115.18%), AUC_0‐t_ (GMR=115.03%), and C_max_ (GMR=101.89%) fell within the bioequivalence limits (80% to 125%) (Table 1). These results indicate that food has no effect on the extent of ACP‐204 absorption, and minimal effect on the absorption rate, which was slightly delayed (∼3 h) under fed (T_max_=8.98 hours) vs fasted (T_max_=5.98 hours) conditions. There were 4 (11.1%) drug‐related TEAEs, with no serious TEAEs and no AEs leading to death or discontinuation. No differences in safety were observed between fasted and fed states.

**Conclusion:**

The criterion for bioequivalence for fed vs fasted was within the bioequivalence boundary for C_max_ and AUC, indicating that food has no meaningful effect on the rate or extent of ACP‐204 absorption. A single 60‐mg dose of ACP‐204 was safe and generally well tolerated, with no unexpected safety findings.